# Hypertension Diagnosis Index for Discrimination of High-Risk Hypertension ECG Signals Using Optimal Orthogonal Wavelet Filter Bank

**DOI:** 10.3390/ijerph16214068

**Published:** 2019-10-23

**Authors:** Jaypal Singh Rajput, Manish Sharma, U. Rajendra Acharya

**Affiliations:** 1 Department of Electrical Engineering, Institute of Infrastructure, Technology, Research and Management (IITRAM), Ahmedabad 380026, India; jaypalsingh@gmail.com; 2 Department of Electronics and Computer Engineering, Ngee Ann Polytechnic, Singapore 599489, Singapore; aru@np.edu.sg; 3 Department of Biomedical Engineering, School of Science and Technology, Singapore University of Social Sciences, Singapore 599494, Singapore; 4 International Research Organization for Advanced Science and Technology (IROAST), Kumamoto University, Kumamoto 860-8555, Japan

**Keywords:** hypertension, ECG, wavelets, optimization, semidefinite program, filter design

## Abstract

Hypertension (HT) is an extreme increment in blood pressure that can prompt a stroke, kidney disease, and heart attack. HT does not show any symptoms at the early stage, but can lead to various cardiovascular diseases. Hence, it is essential to identify it at the beginning stages. It is tedious to analyze electrocardiogram (ECG) signals visually due to their low amplitude and small bandwidth. Hence, to avoid possible human errors in the diagnosis of HT patients, an automated ECG-based system is developed. This paper proposes the computerized segregation of low-risk hypertension (LRHT) and high-risk hypertension (HRHT) using ECG signals with an optimal orthogonal wavelet filter bank (OWFB) system. The HRHT class is comprised of patients with myocardial infarction, stroke, and syncope ECG signals. The ECG-data are acquired from physionet’s smart health for accessing risk via ECG event (SHAREE) database, which contains recordings of a total 139 subjects. First, ECG signals are segmented into epochs of 5 min. The segmented epochs are then decomposed into six wavelet sub-bands (WSBs) using OWFB. We extract the signal fractional dimension (SFD) and log-energy (LOGE) features from all six WSBs. Using Student’s *t*-test ranking, we choose the high ranked WSBs of LOGE and SFD features. We develop a novel hypertension diagnosis index (HDI) using two features (SFD and LOGE) to discriminate LRHT and HRHT classes using a single numeric value. The performance of our developed system is found to be encouraging, and we believe that it can be employed in intensive care units to monitor the abrupt rise in blood pressure while screening the ECG signals, provided this is tested with an extensive independent database.

## 1. Introduction

High blood pressure or hypertension (HT) is a severe disease, and patients have no symptoms in the early stages. Due to low awareness and without proper treatment, this may result in it being more harmful for hypertensive patients and increases the possibility of having cardiovascular diseases. In today’s world, due to hypertension, the number of deaths has increased [1]. As per the 2005 global data, in India, 20.6% of males and 20.9% of females were suffering from hypertension. This trend is expected to rise to 22.9% (male) and 23.6 (female)%. The current survey shows the pervasiveness of hypertension in rural and urban India to be 25% and 10%, respectively. Only 25% of hypertension patients have their blood pressure (BP) under control after the treatment [2]. The BP is the pressure exerted by the blood against the walls of the arteries. The pressure relies on the work being done by the heart and the obstruction of the blood vessels [2]. The possible reasons for hypertension are less physical activity, lifestyle, smoking, stress, family history, and kidney disease [1]. Hence, it is a crucial issue to develop awareness, medical care, and treatment for hypertension. Clinically, we can classify hypertension into mild, moderate, and severe states [3]. It is more important to identify the severity of hypertension. The ranges of normal and hypertension blood pressure are given in Table 1.

The electrocardiogram (ECG) is a valuable tool used to measure the electrical activity of the heart [4,5,6,7,8,9]. Currently, there are various wearable and non-intrusive devices used to monitor hypertension using ECG [10,11,12]. To diagnose hypertension in a clinical environment, blood pressure measurement, which is the gold standard, is used. Depending on the range of blood pressure values (Table 1), the patients are classified as low-risk hypertension (LRHT) and high-risk hypertension (HRHT) patients.

Various techniques, algorithms, applications, and devices have been developed to detect and monitor hypertensive patients. Voss et al. [13] used high-resolution ECG, heart rate variability (HRV), blood pressure variability (BPV), and baroreflex sensitivity (BRS) signals. They found a difference in HRV signals of a normal pregnant female and a hypertensive pregnant female.

Poddar et al. [14] used the automated classification of hypertension and coronary artery disease patients using the probabilistic neural network (PNN), k-nearest neighbor (KNN), and support vector machine (SVM) classifiers with HRV analysis. They obtained the highest classification accuracy of 96.67%. Natrajan et al. [15] observed a significant reduction in high-frequency and an increase in low-frequency HRV signals of hypertensive patients.

Melillo and Izzo [16] used HRV signals along with various machine learning algorithm (SVM, decision tree (DT), and convolution neural network (CNN)) to identify HRHT patients, and obtained the highest accuracy of 87.8%.

Recently, Ni and Wang used fine-grained HRV-based methods and obtained an accuracy of 95% [3]. Song et al. [17] classified normal, hypertensive, and coronary heart disease (CHD) patients using HRV signals and the naive Bayes classifier. They obtained 92.3% classification accuracy.

Yue et al. [18] used machine learning algorithms to implement an automatic risk indication for mask-hypertension using HRV analysis [18]. They found that HRV, parameters in essential hypertension (EH), and mask hypertension (MH) in patients have significantly decreased.

Ni et al. [10] studied hypertension patients and normal subjects using a three-dimensional feature method with continuous HRV monitoring. They obtained the highest classification accuracy of 93.33% [10]. Mussalo et al. [19] analyzed different HRV features for various stages of hypertension patients. They observed significant changes in the HRV parameters of hypertension patients.

Thus, all the above-mentioned studies used HRV signals derived from ECG. The novelty of the proposed work is that we use optimal wavelet-based features extracted from ECG signals instead of using HRV. The optimal orthogonal wavelets that are designed by optimizing spectral localization (SL) were used in the proposed study [20]. Wavelets are regarded as the best tools for the analysis of non-stationary signals, including ECG [21,22,23,24]. Hence, we employed wavelet-based ECG features to develop an automated system for the identification of LRHT and HRHT. We applied the SL-optimized wavelet filter due to the following reasons [25]: (i) In conventional methods, most of the studies were performed by optimizing stop-band and pass-band energies by accurately defining the edge frequencies [26]. That may not be understood a priori in each application. Here, we used the orthogonal wavelet filter [27,28,29] designed by minimizing its frequency spread. (ii) Minimizing the spectral localization of a filter, it is possible to take care of both ripples and the transition band of the filter. (iii) The SL optimized filter gives precisely fewer ripples and sharp roll-off [30].

In our study, we used the SL optimal OWFB. Using a semi-definite program (SDP) technique [31,32], we optimized the filter coefficients, and the interior point algorithm provided the optimized solutions [33,34,35]. Hence, we tested the optimized OWFBs for analyzing ECG signals in order to separate LRHT from HRHT patients. The OWFB provided various sub-bands (SBs) of the ECG signal, and from these SBS, we extracted log energy (LOGE) and spectral fractal dimension (SFD) features [36]. Student’s *t*-test ranking was applied to all extracted features and the most significant SBs of SFD and LOGE features.

The main contribution of this study is the development of the hypertension diagnosis index (HDI) using OWFB-based SFD and LOGE features. HDI provides the discrimination of LRHT and HRHT by a single numeric value. In a clinical environment, HDI is simpler and easier to use for the diagnosis of disease.

The remainder of the paper is arranged in the following manner. We discuss the details of the ECG dataset in Section 2. The methodology and an optimally-designed OWFB are described in Section 3. Section 4 illustrates the results obtained. In Section 5, we discuss the results obtained. At last, the concluding remarks of the paper are outlined in Section 6.

## 2. Dataset

**Figure 1:**
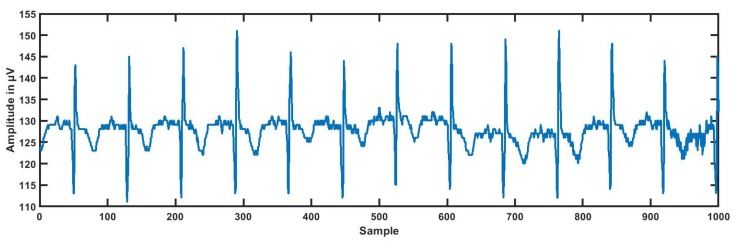
Typical low-risk hypertension (LRHT) ECG signal.

**Figure 2:**
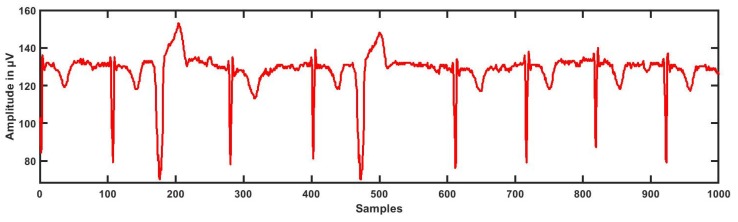
Typical high-risk hypertension (HRHT) myocardial infraction ECG signal.

**Figure 3:**
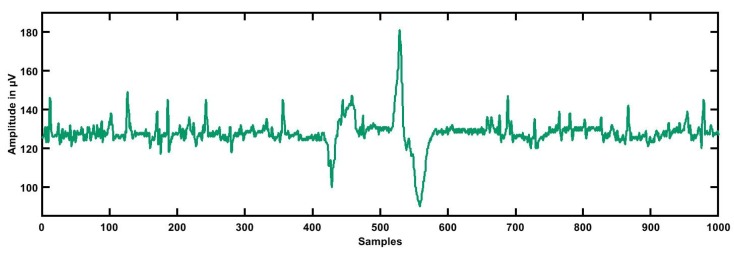
Typical high-risk hypertension (HRHT) syncope ECG signal.

**Figure 4:**
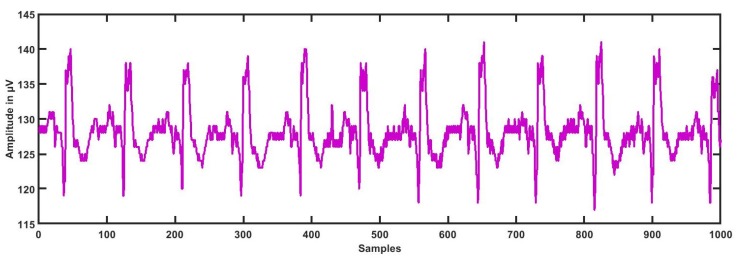
Typical high-risk hypertension (HRHT) stroke ECG signal.

The dataset for this study was taken from physionet’s smart health for assessing the risk of events of ECG signals (SHAREE project) https://archive.physionet.org/pn6/shareedb/. A total of 139 subjects’ ECG recordings were used with a length of 2 h:10 min:12 s, approximately, for each ECG signal. Each ECG recording contained three rows (channels/ signals) III, V3, and V5, and each signal had 1 million samples, approximately. In our study, leads III, V3, and V5 were named CH1 (Channel 1), CH2, and CH3, respectively. The ECG sampling frequency was 128 Hz with an 8-bit resolution, and the sampling interval was 0.0078125 s. The average age of patients was 71.4 for LRHT and 74.5 years for HRHT, which included 49 female and 90 male patients. To detect major cardiovascular and cerebrovascular events, patients were observed for a year. Seventeen patients (three syncopal, three strokes, eleven myocardial infarctions) were identified as HRHT subjects, while 122 patients as LRHT subjects. The dataset was authorized by the Federico II University Hospital Trust’s Ethics Committee. All subjects involved in data collection provided consent and signed for the experimental use of data. Table 2 gives the details and statistics of the patients included in gathering the SHAREE database. We segmented our ECG signal into 5-min signals. After the segmentation of ECG signals, we obtained 3172 ECG epochs corresponding to LRHT and 442 epochs for HRHT subjects for each channel. Figure 1, Figure 2, Figure 3 and Figure 4 show the LRHT and HRHT ECG signals of 5 min.

## 3. Methodology

**Figure 5:**
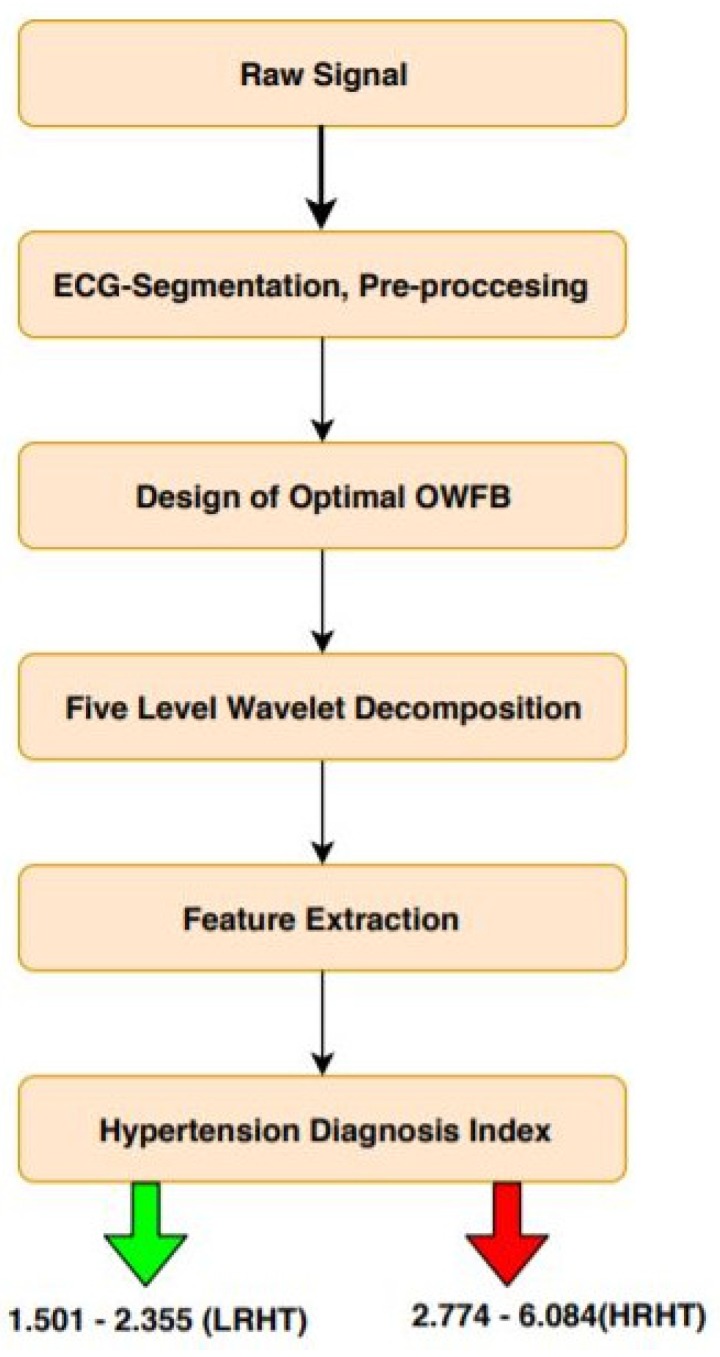
Workflow of the proposed work. OWFB, optimal orthogonal wavelet filter bank.

To separate LRHT and HRHT automatically, the optimally-designed OWFB was used. A total of six sub-bands (SBs) were produced using wavelet decomposition [26]. Five SBs were used for detail, and one SB for approximation was used for ECG signals. After the wavelet decomposition into six SBs’ log energy (LOGE) and signal fractional dimensions (SFD), features were extracted from all SBs. Thus, a total of 12 features were obtained from each ECG epoch, six LOGE and six SFD. The novelty of this work is the development of the HDI, which can be used to discriminate LRHT and HRHT ECG signals. The detailed outline of the proposed automated high-risk hypertension detection system is shown in Figure 5.

### 3.1. ECG Segmentation

For fast computing, we pre-processed the ECG signals. Long duration (2 h:10 min:12 s) ECG data were segmented for the 5-min duration, then each ECG segment was normalized using the Z-score before applying to the wavelet filter bank.

### 3.2. Design of Filter Bank

**Figure 6:**
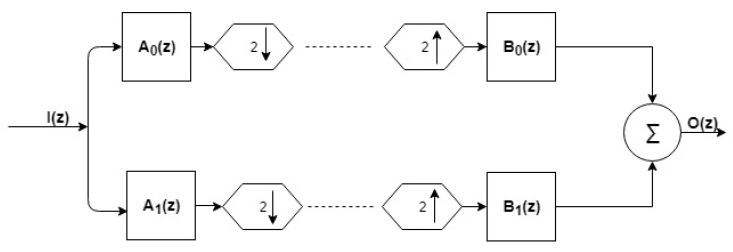
Two-channel OWFB.

The OWFB contained two sets of filter banks (FLBs) (Figure 6); one was called synthesis FLB, and another one was analysis FLB [25]. Both FLBs contained low-pass (LP) and high-pass (HP) filters. For analysis FLB, the outputs of LP and HP were downsampled by 2, and in synthesis FLB, the inputs to HP and LP were up-sampled by a factor of 2 prior to applying them. Let $A_{0}\left(z\right)$ and $A_{1}\left(z\right)$ be the LP and HP filters, respectively, for analysis FLB. Let $B_{0}\left(z\right)$ and $B_{1}\left(z\right)$ be the LP and HP filters of synthesis FLB, as shown in Figure 6. The analysis and synthesis LP filters were time-reversed copies of each other, which is an important characteristic of OWFB [20]. Using the quadrature conjugation technique [37], the HP filters $A_{1}\left(z\right)$ and $B_{1}\left(z\right)$ can be derived from LP filters $A_{0}\left(z\right)$ and $B_{0}\left(z\right)$. Hence, we can get the remaining three filters directly from the LP analysis filter $A_{0}\left(z\right)$. The perfect reconstruction (PR) and zero-moment (ZM) constraints [28] must be obeyed by optimal OWFB for output O(z) to be a delayed replica of the input I(z) [29].

For perfect reconstruction, the filter must fulfill the orthogonality condition as mentioned below [25]:

(1) $$A_{0}\left(z\right)B_{0}\left(z\right)+A_{0}\left(-z\right)B_{0}\left(-z\right)=2$$

Let $P\left(z\right)=A_{0}\left(z\right)B_{0}\left(z\right)$, P(z) be called the product filter [34]. We can rewrite (1) in terms of the product filter as below:

(2) P(z)+P(−z)=2

Let $P(e^{jf})$ be the frequency response of the product filter, which is represented by [34]:

(3) $$P\left(e^{jf}\right)=\Sigma_{n}P\left(n\right)e^{-jfn}$$

Now, we can represent the perfect reconstruction condition (2) in the frequency domain as,

(4) $$|P\left(e^{jf}\right){|}^{2}+|P(e^{j(\pi -f)}{|}^{2}=2$$

For the real $A_{0}\left(n\right)$, $P\left(e^{jf}\right)={\left|A_{0}\left(e^{jf}\right)\right|}^{2}\geq 0$ [38]. Here, to design a real-coefficient orthogonal wavelet filter bank, a positive value of the $P(e^{jf})$ for f∈[0,π] is needed. The total number of roots at z=−1 can be defined as zero moments (ZM) of the filter [29,39]. To design the LP filter with $M^{th}$-order, ZMs there should be 2M zeros at z=−1 in the product filter.

If the product filter satisfies Equation (2) and the $P(e^{jf})\geq 0$, f∈[0,π] condition, we can convert the designed OWFB into the designed product filter P(z) [40,41,42]. After designing P(z), we can obtain the required analysis LP filter $A_{0}\left(z\right)$ using spectral factorization [35,43].

#### Constraint in the Time-Domain and Objective Function

To design the optimal OWFB, consider a(n) to be the unit impulse response of finite impulse response analysis LP filter of $A_{0}\left(z\right)$ and b(n) be the unit impulse response of synthesis LP filter $B_{0}\left(z\right)$ of order N−1. The optimality criterion to design the orthogonal filter design is to minimize the mean squared spectral localization (MSSL). MSSL happens to be the same for both analysis and synthesis filters as the former is the time-reversed replica of the latter [24,32,44].

Now, we can define the MSSL, ${\sigma_{f}}^{2}$ of the filter $A_{0}\left(z\right)$ as [45]:

(5) $${\sigma_{f}}^{2}=\frac{1}{2\pi E}\int_{-\pi }^{\pi }f^{2}{|A_{0}\left(e^{jf}\right)|}^{2}df$$

where *E* represents the squared-norm or energy of the filter. Imposing *M* zero-moments (regularity) and orthogonality constraints, we design an optimal OWFB with the objective of having minimum MSSL. The optimization problem for the filter design can be mentioned as below.

(6a) $$\begin{array}{cc} \; & \underset{a[n]}{min}\;\;{\sigma_{f}}^{2}=\frac{1}{2\pi E}\int_{-\pi }^{\pi }f^{2}{|A_{0}\left(e^{jf}\right)|}^{2}df\\ \; & \mathrm{subject}\;\mathrm{to} \end{array}$$

(6b) $$\begin{array}{cc} \; & \sum_{n=0}^{N}a\left(n\right)a\left(n-2m\right)=\delta \left(m\right);m=0,1,\cdots ,\frac{N}{2}-1 \end{array}$$

(6c) $$\begin{array}{cc} \; & \sum_{m=0}^{N}{\left(-1\right)}^{m}m^{k}a\left(m\right)=0;k=0,1,\cdots ,M-1 \end{array}$$

Hence, to minimize MSSL (6a) for the given regularity (6c) and orthogonality (6b) constraints, a constrained optimization problem was formulated. To develop a convex formulation, we need to express the constraints and objective function in terms of P(z). As specified above, the sequence p(n) (impulse response of the P(z)) is an auto-correlation sequence whose spectrum satisfies the non-negativity condition $P(e^{jf})\geq 0$. We can write the objective function (5) in the form of the product filter as:

(7) $${\sigma_{f}}^{2}=\frac{1}{2\pi E}\int_{-\pi }^{\pi }f^{2}\left|P(e^{jf})\right|df=\frac{1}{\pi E}\int_{0}^{\pi }f^{2}\left|P(e^{jf})\right|df$$

Thus, the optimization problem (6) can be represented in the form of the product filter as mentioned below:

(8a) $$\begin{array}{cc} \; & \underset{p\left[m\right]}{min}\frac{1}{\pi }\int_{0}^{\pi }f^{2}P\left(e^{jf}\right)df\\ \; & \mathrm{subject}\;\mathrm{to} \end{array}$$

(8b) $$\begin{array}{cc} \; & p\left[2m\right]=\delta \left(m\right);m=0,1,\cdots ,\frac{N}{2}-1 \end{array}$$

(8c) $$\begin{array}{cc} \; & p\left(0\right)+2\sum_{m=1}^{N-1}{\left(-1\right)}^{m}p\left(m\right)=0 \end{array}$$

(8d) $$\begin{array}{cc} \; & \sum_{m=0}^{N-1}{\left(-1\right)}^{m}m^{2k}p\left(m\right)=0;k=0,1,\cdots ,M-1 \end{array}$$

(8e) $$\begin{array}{cc} \; & P(e^{jf})\geq 0;f\in \left[0,\pi \right] \end{array}$$

The above-mentioned optimization problem (8) is a non-convex optimization problem in variable p(n), whereas the optimization problem in (6) is a convex optimization problem in variable a(n). Now, we intend to convert the non-convex problem into a convex problem to get an optimal solution. Using (8b), the half-band condition is linear in variable p(n). Equations (8c) and (8d) represent the regularity conditions, which are also linear constraints. Only the non-positivity condition (8e) is a non-linear semi-infinite constraint (one constraint each for every f∈[0,π]), which needs to be converted into a finite constraint to formulate the convex optimization problem. Sharma and Moulin et al. [46,47] used the discretization method to transform the semi-infinite constraint into finite constraints. However, due to the inaccurate solution obtained by the discretization method, it is not advisable to use it.

Hence, we used the Kalman–Yakubovich–Popov lemma (KYPL) [48] for the formulation of a semidefinite program (SDP). By the KYP lemma, (8e) exists only if there exists a symmetric positive $Z\in R^{N\times N}$ such that:

(9) $$p\left(m\right)=\sum_{n}{\left[Z\right]}_{n,n+m};m=0,1,\cdots ,N-1$$

Hence, the objective function (8a) in terms of sequence p(n) can be given below:

(10) $$\sigma_{f}^{2}=\frac{\pi^{2}}{3}p\left(0\right)-\sum_{m=0}^{\frac{N-2}{2}}\frac{4p(2m+1)}{{\left(2m+1\right)}^{2}}={\mathrm{sp}}^{T}$$

Here, $s\in R^{N}$ is $\left[\frac{\pi^{2}}{3},\frac{-4}{1^{2}},\frac{-4}{3^{2}},0,\cdots ,0,\frac{-4}{{\left(N-1\right)}^{2}}\right]$ and $p\in R^{N}$ is [p(0),p(1),⋯,p(N−1)]. Using (10), we obtained the objective function as a linear function of p(n). Furthermore, all constraints can be expressed as a linear function of p(n). Hence, the optimization problem (8) can be written as the following convex optimization problem [25].

(11) $$\begin{array}{c} \underset{Z⪰0,p(m)}{min}\sigma_{f}^{2}={\mathrm{sp}}^{T}\\ \mathrm{subject}\mathrm{to}\;(8b),(8c),(8d),(9)\;\mathrm{and}\;Z≻0 \end{array}$$

Now, our optimization problem is convex as the objective function, and all the constraints are convex. To find the global solution of the problem, we can use interior point algorithms such as SedDumiand SPDT3 [49].The tools SPDT3 and SedDumi can solve the optimization problem accurately and efficiently. After finding the optimal p(n), the next step is to obtain the desired low-pass filter $A_{0}\left(z\right)$ using spectral localization of P(z).

### 3.3. Wavelet Decomposition

We designed optimal WFBs for sub-band decomposition of ECG signals [50,51]. We employed five levels of decomposition. The five-level wavelet decomposition gave us precise information about the 6 SBs of each ECG epoch. By this technique, we have extracted the desired frequencies present in the ECG signal. Hence, the five-level wavelet decomposition of the ECG signal was done by this method. The six SBs had five detailed (SB1–SB5) and one approximate (SB6) SBs.

### 3.4. Features Used

The selection of essential features was an important part of this work. Using this method, we were able to classify LRHT and HRHT ECG signals. The log energy (LOGE) and signal fractal dimension (SFD) features were computed from all six SBs.

Log energy (LOGE): To calculate the LOGE of each SB of the ECG signal, the logarithm of energy needs to be computed. The general formula of the log energy is [25]:

(12) $$LOGE_{m}=log\sum_{n}{\left|r_{m}\left(n\right)\right|}^{2}$$

where $LOGE_{m}$ is the log energy of the $m^{\mathrm{th}}$ sub-band and $r_{m}\left(n\right)$ is the amplitude of the $n^{\mathrm{th}}$ sample of the $m^{\mathrm{th}}$ sub-band.

Signal fractal dimension (SFD): Fractals are figures of geometry or curves that are a subset of a Euclidean space. These curves have the Hausdorff dimension strictly exceeding the topological dimension. The fractal dimension provides a statistical magnitude to the complexity detailing the pattern or fractal pattern with respect to the scale with which it is measured [25].

We can write the SFD equation as below:

(13) $$SFD=\frac{log(P_{m})}{log(\frac{1}{m})}$$

where $P_{m}$ is the number of self-similar patterns used to fill the original pattern and *m* is the ratio used to decompose the original pattern into $P_{m}$ self-similar patterns.

### 3.5. Hypertension Diagnosis Index

The extracted highly-significant features in Table 3 were used to develop the mathematical model (14) to discriminate the two classes [52,53,54].

We propose a hypertension diagnosis index (HDI) to discriminate against the LRHT and HRHT by a single numeric value. We used two sets of features (SFD and LOGE) with the highest *t*-value (lowest *p*-value) to compute the HDI [55]. We formed the mathematical simulation given by:

(14) $$\begin{array}{c} HDI=6-\left(3\times LOGE_{SB2}+4\times LOGE_{SB3}+SFD_{SB6}\right)-15\times (SFD_{SB2}+SFD_{SB3}+SFD_{SB4}) \end{array}$$

where $LOGE_{SB2}$ and $LOGE_{SB3}$ represent LOGE features extracted from SB2 and SB3, while $SFD_{SB6}$, $SFD_{SB2}$, $SFD_{SB3}$, and $SFD_{SB4}$ represent the SFD feature extracted from SB6, SB2, SB3, and SB4.

## 4. Results

Using the 5-min ECG dataset, it is segmented in 3172 epochs of low-risk hypertension and 442 epochs of high-risk hypertension. Our whole experimental work was performed using MATLAB Version 9.1 with an Intel Xeon 3.5 gigahertz (GHz) and 16 gigabytes (GB) of random access memory (RAM). Table 3, Table 4 and Table 5 represent the statistical differences of LRHT and HRHT for the CH3, CH1, and CH2 ECG signals.

**Figure 7:**
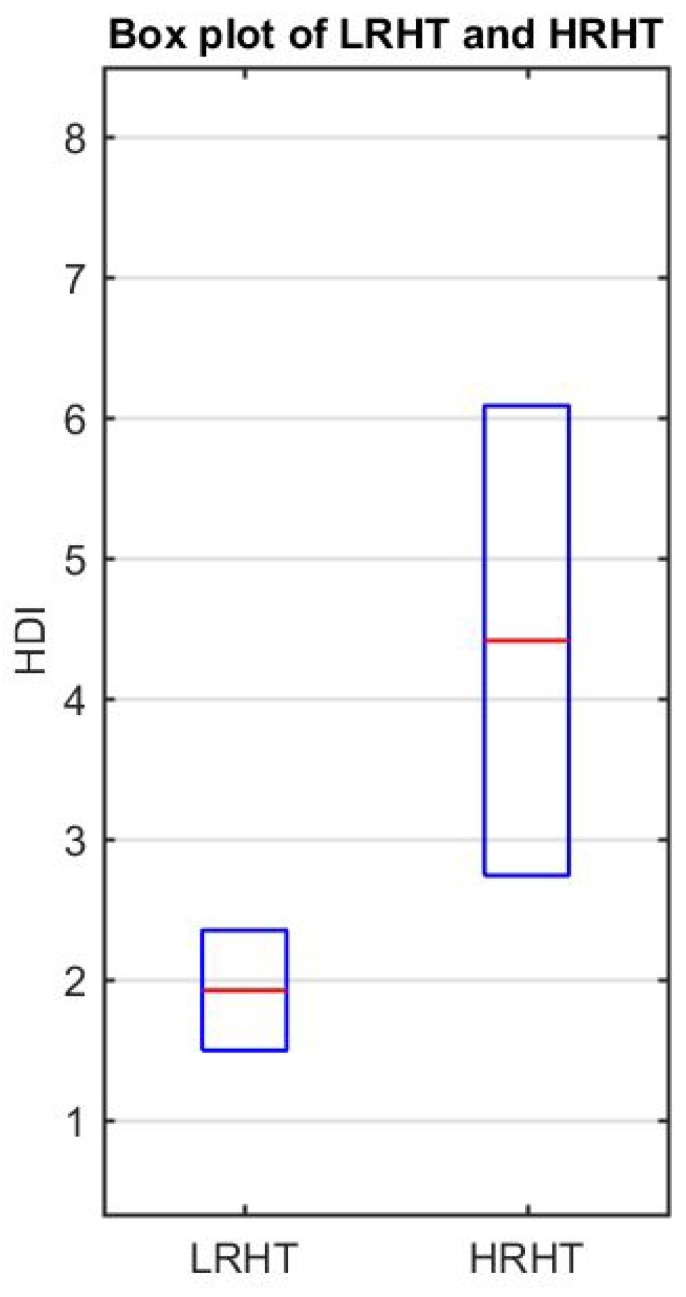
Box plot of LRHT and HRHT.

The result of Student’s *t*-test for each SB is shown in Table 6 with *t*-values and *p*-values for both features (SFD and LOGE). Table 7 shows the result of the calculated HDI. Table 7 presents the range of HDI and shows a significant difference in the LRHT and HRHT. Figure 7 shows the discrimination between LRHT and HRHT by the significant numeric value. The segregation of both classes by HDI is more simple and easy to use in a clinical environment.

## 5. Discussion

The aim of this study was to calculate the performance of features (LOGE and SFD) extracted from novel optimal OWFB. In this research work, using spectrum-localized OWFB-based non-linear features, we could identify HRHT patients using a single numeric value. The employed optimal OWFB-based features yielded 100% discrimination of LRHT and HRHT patients using HDI. The salient features of our developed automated system are given below:

- From Table 3, LOGE values of SB2–SB6 showed significant changes corresponding to LRHT and HRHT patients.
- SFD of SB2 for LRHT and HRHT obtained the highest mean value, while SB1 showed the lowest mean value. LOGE of SB1 yielded the highest mean value for LRHT, and SB2 for HRHT patients yielded the lowest mean value.
- The novelty of the proposed work was the development of HDI to discriminate between the two classes using a single value.
- Table 7 presents the range of HDI and shows a significant difference in the LRHT and HRHT by a numeric value.
- We did not need classifiers, which involve training and testing. It was fast and involved only the extraction of two feature sets.
- The spectral localization technique was used to analyze non-stationary characteristics of the ECG signal. As we used spectral localized OWFB, our proposed work was unique as compared to other research works [3,16].
- For better and fast computation, we used fewer features. The length of the ECG signal was 5 min. Hence, it was not computationally intensive and quicker in diagnosis.
- Using the same database, Melillo and Izzo used various machine learning algorithms (SVM, decision tree (DT), and convolution neural network (CNN)) and obtained the highest accuracy of 87.8% with HRV signals [16]. Recently, Ni and Wang [3] obtained an accuracy of 95% using heart rate variability (HRV) signals.
- Many studies have used HRV-based techniques to detect hypertension; we used wavelet-based features directly extracted from ECG. Our method was different from HRV-based methods and easy to use in the clinical environment [3].
- The performance of the system was found to be promising, and we expect that it can be employed in intensive care units to monitor the abrupt rise in blood pressure while screening the ECG signals, provided it is tested with an extensive independent database.
- The present research work was conducted using 139 ECG recordings segmented into 3614 (3172 as LRHT, 442 as HRHT (78 stroke, 78 syncope, and 286 myocardial infarction)) epochs of 5 min each comprised of CH1, CH2, and CH3. The ECG dataset was obtained from https://archive.physionet.org/pn6/shareedb/. Our whole experimental work was performed using MATLAB. Table 7 shows the results of the automated detection of LRHT and HRHT classes. In Table 8, we compare our proposed work with other methods. Using HDI, we can discriminate between the two classes by just the single numeric value with 100% accuracy.

The dataset consisted of 3614 ECG epochs, out of which 87% were LRHT and 13% were HRHT ECG signals. This imbalanced dataset is one of the limitations of our work. In general, LRHT data are greater than HRHT data. To reduce this imbalance problem, synthetic balancing data are needed. The other limitation of our research work is the selection of the optimal number of ZMs and the length of the filter. In order to achieve accurate identification of HRHT, we cannot predict the estimated order and ZM a priori.

In recent studies, deep learning methods were widely used for classification problems [56,57,58,59]. We can use deep learning methods like convolution neural networks (CNN) [60]. In deep learning-based techniques, we need not extract, select, and classify the handcrafted features. However, due to extensive data processing, the computational complexity is enormous. Hence, they require fast processing workstations and graphics processing units (GPU).

## 6. Conclusions

In this study, we used optimal OWFB-based non-linear features to discriminate LRHT and HRHT ECG signals automatically using an index (HDI). The five-level wavelet decomposition of ECG signals using optimal OWFB produced six (SBs). The LOGE and SFD features were extracted for all six SBs. Our proposed OWFB-based method was adequate to discriminate the HT ECG signals accurately utilizing features (LOGE and SFD) by a single numeric value. To evaluate the performance of the optimal wavelet filter bank, HDI was developed, which separated LRHT and HRHT groups using the proposed index. Our results show that the developed model was better than the other existing systems and ready to be tested using a large database. In the future, we plan to test the performance of our technique to detect the severity of hypertension using certain machine learning-based techniques with the same database. We also intend to use deep learning-based methods for the classification of LRHT and HRHT ECG signals as our future work.

## Figures and Tables

**Table 1:** Typical blood pressure ranges [3].

Blood Pressure Category	Systolic (mmHg)	Disystolic (mm Hg)
Normal BP	less than 120	less than 80
Elevated, Normal Hypertension	120–129	less than 80
Stage 1	130–139	80–89
High-risk Hypertension		
Stage 2	greater than 140	greater than 90
High-risk Hypertension		
Stage 3	greater than 180	greater than 120
High-risk Hypertension		

**Table 2:** Statistics of the patients employed in acquiring the database. LRHT, low-risk hypertension; HRHT, high-risk hypertension.

S.no	Parameters	LRHT Class		HRHT Class	
		Mean	Standard Deviation	Mean	Standard Deviation
1	DBP	76.31	9.1	73.5	8.4
2	SBP	136.6	19.5	141.7	23.5
3	BMI	27.6	3.9	27.9	4.9
4	LVMi	130	26.1	140.2	25.1
5	Age in years	71.4	7	74.1	6.5

DBP = diastolic blood pressure, SBP = systolic blood pressure, BMI = body mass index, and LVMi = left ventricular mass index.

**Table 3:** Mean and standard deviation for CH3.

Sub Bands	SFD				LOGE			
	LRHT		HRHT		LRHT		HRHT	
	Mean ± Std		Mean ± Std		Mean ± Std		Mean ± Std	
SB1	1.016	0.0032	1.016	0.0031	20.263	0.009	20.263	0.0053
SB2	2.011	0.0217	2.017	0.0252	11.983	0.915	11.711	0.7576
SB3	1.892	0.0217	1.899	0.0260	12.481	0.991	12.128	0.8576
SB4	1.621	0.0328	1.626	0.0395	13.096	0.981	12.746	1.0144
SB5	1.214	0.0210	1.213	0.0211	12.943	1.003	12.602	1.0966
SB6	1.059	0.0077	1.056	0.007	12.821	1.089	12.565	1.2976

**Table 4:** Mean and standard deviation for CH1. SB, sub-band.

Sub Bands	SFD				LOGE			
	LRHT		HRHT		LRHT		HRHT	
	Mean ± Std		Mean ± Std		Mean ± Std		Mean ± Std	
SB1	1.024	0.0025	1.025	0.0029	20.263	0.005	20.263	0.0052
SB2	2.012	0.0231	2.020	0.0272	12.278	0.713	12.122	0.7550
SB3	1.898	0.0212	1.901	0.0223	12.564	0.858	12.298	0.7611
SB4	1.634	0.0348	1.641	0.0288	13.172	0.796	12.912	0.7564
SB5	1.212	0.017	1.213	0.0179	13.057	0.838	12.794	0.8087
SB6	1.065	0.0039	1.064	0.0035	12.857	0.911	12.673	1.0303

**Table 5:** Mean and standard deviation for CH2.

Sub Bands	SFD				LOGE			
	LRHT		HRHT		LRHT		HRHT	
	Mean ± Std		Mean ± Std		Mean ± Std		Mean ± Std	
SB1	20.26	0.0146	20.26	0.0106	1.0241	0.003	1.0243	0.0024
SB2	11.99	0.9122	11.76	0.8488	2.0213	0.024	2.0256	0.0291
SB3	12.05	0.9504	11.77	1.0436	1.9083	0.024	1.9121	0.023
SB4	12.64	1.1322	12.55	1.2271	1.6456	0.037	1.6261	0.0348
SB5	12.47	1.2679	12.61	1.250	1.2095	0.017	1.2119	0.0144
SB6	12.34	1.305	12.46	1.2807	1.0644	0.004	1.0643	0.0037

**Table 6:** Student’s *t*-test results, *t*-value and *p*-value. SFD, signal fractional dimension; LOGE, log-energy.

Rank	Feature	*t*-Value	*p*-Value
1	SFD SB6	8.854	9.47$\times 10^{-18}$
2	LOGE SB3	7.943	9.35$\times 10^{-15}$
3	LOGE SB2	6.878	1.45$\times 10^{-11}$
4	LOGE SB4	6.829	2.22$\times 10^{-11}$
5	LOGE SB5	6.196	1.14$\times 10^{-9}$
6	SFD SB2	5.744	1.54 $\times 10^{-8}$
7	SFD SB3	4.982	8.59 $\times 10^{-7}$
8	LOGE SB6	3.952	8.79 $\times 10^{-5}$
9	SFD SB4	2.691	0.007341
10	SFD SB5	1.261	0.207762
11	LOGE SB1	1.009	0.313201
12	SFD SB1	0.958	0.338084

**Table 7:** Hypertension diagnosis index (HDI) range for the LRHT and HRHT classes.

Index	LRHT	HRHT	*p*-value
HDI	1.501–2.355	2.774–6.084	<0.01

**Table 8:** Comparison of work done for automated detection of hypertension ECG signals. HRV, heart rate variability.

**Authors** **(Year)**	**Features and Classifier**	**Classification (in %)**
Simjanoska et al. [61] (2018)	**ECG-based Features Extracted:**	
	• SFD, Entropy	ACC: 96.8%
	**Classifiers:**	
	• SVM	
	• KNN	
Sau et al. [62] (2018)	**Features:**	
	• BMI, Age, Job	ACC: 82.4%
	**Classifiers:**	Spec: 81.5%
	• Random Forest	
	• Tree Based	Pres: 84.6%
Seidler et al. [63] (2019)	**Features Extracted:**	AUC: 0.87%
	• Pulmonary Artery Pressure	ACC: 95%
	**Classifiers:**	
	• SVM	
	• Tree Based	
	• Logistic Regression	
Poddar et al. [14] (2019)	**Features Extracted:**	ACC: 96.7%
	• HRV Linear and Nonlinear	
	**Classification Method:**	
	• Support Vector Machine	
Song et al. [17] (2015)	**Features Extracted:**	ACC: 92.3%
	• HRV in Time Domain	
	• HRV in Frequency Domain	
	• HRV Nonlinear Analysis	
	**Classification Method:**	
	• Naive Bayesian	
Lee et al. [64] (2015)	**Features Extracted:**	ACC: 90%
	• Linear and Nonlinear Features of HRV	
	**Classification Method:**	
	• Support Vector Machine	
Melillo et al. [16] (2015)	**Features Extracted:**	
	• HRV Linear	Spec: 71.4%
	• HRV Nonlinear	Sen: 87.8%
	**Classification Method:**	
	• SVM	
	• Tree-based Algorithm	
	• Artificial Neural Network	
Ni et al. [3] (2019)	**Features Extracted:**	Precision: 95.1%
	• HRV in Time Domain	
	• HRV in Frequency Domain	
	• HRV Nonlinear Analysis	
	**Method:**	
	• Fine-grained Analysis Method	
Presented Work	**Features Extracted of ECG Signal:**	**CH3:**
	• Signal Fractal Dimension	LRHT: 1.501 − 2.355
	• Log-Energy	HRHT: 2.774 − 6.084
	**Method:**	
	• HDI	Proposed Unique Ranges for LRHT and HRHT 100% Separation between Two Classes
